# Supplementary material for: The influence of health facility-level access measures on modern contraceptive use in Kinshasa, DRC
Source: PLoS One. 2020 Jul 23;15(7):e0236018. doi: 10.1371/journal.pone.0236018 (PMC7377448; doi:10.1371/journal.pone.0236018)
Supplement: Attachment — Submitted filename: Comment_PONE-D-19-26324.docx [file pone.0236018.s001.docx]

The paper linked PMA household data and health facility data to assess the association between women’s contraceptive use and different types of access measured at the EA level, as well as other community-level factors aggregated from individual data. The findings could have important implications for family planning programs in Kinshasa, DRC.

Hope the following comments could help strengthen the paper:

Background:

a) Line #51: the statement “the spatial data from the SPA and DHS have been randomly displaced” is not correct. Geolocations of DHS clusters are displaced but not those of health facilities in SPA surveys. The DHS Program released GPS data collected in SPA without displacement.
b) Line #56: I would not say “the SPA and DHS sampling strategy make it impossible to link at the EA level”. Actually, it is possible to link them at the EA level when the SPA survey is a facility census. Please refer to this report for more detail (<https://www.dhsprogram.com/publications/publication-SAR10-Spatial-Analysis-Reports.cfm>)
c) Line #58: I don’t think this recommendation was made in the reference #9. Please double check.
d) Study setting: it would be helpful to include some information on contraceptive method mix in Kinshasa. Do more women use LARCs? This may help interpret the findings on the association that having more facilities with LARCs increases the likelihood of contraceptive use.

Methods:

Conceptual framework: this paragraph reads more like a description of different types of access (i.e. their definitions) rather than a conceptual framework. A conceptual framework would demonstrate/discuss the pathway by which variables, alone or in combination, are associated with the outcome of interest. Also it would be helpful to map the SDP-level variables and other community-level variables introduced later to these different “access” categories.

Data:

a) The sample design of the 4 rounds was independent? Or the same EAs were followed over the 4 rounds? This is not clear.
b) It would be helpful to provide some information on the total number of SDPs in EAs from which the SDP sample were selected. Is it possible that same facilities, such as tertiary hospitals are shared by multiple EAs? If this is the case, how did you deal with this in the analysis?

Key independent variables:

a) SDP variables---I have concerns about the SDP variables constructed at the EA level by counting the total number of SDPs. These include the total number of SDPs that off FP methods, total number of SDPs with more than 3 methods, total number of SDPs with more than 5 methods in stock, and several others. These SDPs were a sample of all SDPs that serve the EA. The total count may not represent the service environment unless you count among all SDPs that serve the EA. Plus you have a max value of 6 for all these indicators, right? Because the sample size of SDPs would not exceed 6 in each EA. Instead of counting, for example the total number of SDPs that offer at least 3 FP methods, a proportion measurement, i.e. proportion of SDPs with at least 3 methods might be a better measurement.

Moreover, in the analysis, do you treat these variables as continuous variable? I guess their possible values would be 0, 1 ,2 ,3 ,4 ,5 ,6? They are not continuous.

b) Community-level variables constructed with individual data --- the decision-making variable is not clear. Decision making for what?

Analysis

a) I assumed you pooled data from 4 rounds for the multivariate analysis. Please make this clear in the description
b) I am concerned about putting all the SDP variables in the same model. Did you check the correlation among them? Some may be highly correlated for example, number of facilities with 3 methods and number of facilities with 5 methods. Others could be highly correlated too. I would double check. I wonder if this is the reason for the lack of significance of many SDP variables.
c) Line #239. It should be noted as formula (3)
d) Line #241: It should not be noted as formula (3)
e) Was the complex survey design accounted for in the analysis? The paper did not mention this.

Results

a) Table 2: what is LAPM? This term appears in several places. Please correct/clarify.
b) Line #343-353: where are the results described in this paragraph?
c) Table 4: I did not see the results on the interaction terms. The authors said something about such results in Line #435. But it is not clear which EA-level variables affect contraceptive use differently in the four rounds.
d) Line 387: the association between contraceptive use and number of facilities with water and electricity was negative not positive (OR<1).
e) The authors often use “x% more likely” in the interpretation of odds ratios. This is not correct. These are odds ratios not probabilities. It should be interpreted as something like this: the odds of using contraception is x% more/higher for group A compared to Group B.
f) I did not see where in the results showed the answers to Research Question 2) and 3). To answer these questions, the authors need to assess the additional variation explained by adding another group of variables, for example, from model 2 to model 3, how much (e.g. %) additional variation is explained by adding other community-level variables.

Discussion

a) Line 473: some references seem to be missing here.
b) The authors may want to discuss a few surprising findings from the multivariate analysis: these include: the negative association between contraceptive use and number of facilities with water and electricity in models 2, 3, and 4, negative association between contraceptive use and number of SDPs that insert implant in model 4.
