# Supplementary material for: The influence of health facility-level access measures on modern contraceptive use in Kinshasa, DRC
Source: PLoS One. 2020 Jul 23;15(7):e0236018. doi: 10.1371/journal.pone.0236018 (PMC7377448; doi:10.1371/journal.pone.0236018)
Supplement: Attachment — Submitted filename: Response to the reviewer.docx [file pone.0236018.s002.docx]

We appreciate the reviewer’s comments to strengthen the presentation of this study. We have responded to the comments and have made the changes as follows:

***Introduction

1- I think the introduction could be shortened by quite a bit.***

- We have revised the introduction to shorten it.

***2- Please provide a reference for the DRC policy stated in Line 63 through 66.***

- Thank you for noticing. We added the citation.

***3- Please provide a reference for the mCPR figures reported in Line 71.***

- We added the citation.

***4- In terms of structure, I believe it is best to begin the manuscript from a broad global perspective and then narrow it down to focus on DRC. As written the manuscript begins by talking about the specific goals of the DRC in the context of its African subregion. Then in the third paragraph (Line 75 through 84) the authors suddenly move back to the global objectives regarding fertility and family planning. Much of that information in that paragraph could also be condensed to a few lines. In the interest of keeping the introduction a bit shorter, I think the authors can reference those global mobilization efforts and mention that they have influenced the DRC’s specific policies, as opposed to devoting one paragraph to them.***

- We revised the introduction to follow the recommended order.

***5- It is best to provide the full name of the meeting as opposed to simply describing it as the London Summit (Line 76)***

- We edited the name. However, we decided to delete that sentence to shorten the introduction.

***6- I believe the authors can also shorten the two paragraphs that situate the idea of access (Line 86 - 91) and measuring access (Line 93 through 109). Perhaps those two paragraphs could be consolidated as well. For example, in the paragraph that discusses measuring access and the tools available;***

- We have revised the mentioned paragraph to be more concise.

***7- I feel the conversation abut the limitations of DHS and SPA data can be saved for the discussion. Simply focus the paragraph on the advantage of using***

- We believe that the difference between the previous instruments and surveys to measure access and PMA2020 is important. However, in our discussion we tried to emphasize the significance of the results of current study in the context of the DRC rather than discussing the survey. In addition, Choi et al. have published an article using DHS to measure access and have discussed the shortcomings of the instrument.

***8- The last paragraph (Line 128 - 138) is not necessary, especially after the manuscript’s broad objectives and aims have stated previously.***

- We deleted the paragraph.  ***Methods***

***9- It is a good idea to have a couple of sentences to explain the choices of the constructs in the methodological framework perhaps tying it back to the idea of an inadequate definition of access and the difficulties associated with measuring access. I am asking for some justification in choosing those six constructs (Line 144). Also, the placement of that paragraph seems odd, given that it has no connection to the measurements section of the paper which is what the conceptual framework serves.***

- We have adopted the framework from previous relevant publications on frameworks of access to family planning service (Bertran et al. 1994 and Choi et al. 2016)

***10- I am not sure the data subsection should be used for contrasting DHS and SPA surveys with the survey design of PMA2020. Really this subsection should focus on describing PMA2020 and its design. It may also be relevant to state or highlight the partnership that led to the PMA2020 surveys.***

- We edited the paragraph to follow your recommendation. ***Results***

***11- The grammar is a bit muddled in the initial reports of findings. The authors report certain figures as if they were looking at cross-sectional data as opposed to multiple waves of data. For example, Line 333, the authors mention that “Women had 1.7-1.8 live children on average at the time of the interview”. When I think they mean to say that across the four waves, average live child ranged from 1.7 to 1.8. Be clear that those statistics reported range over the different rounds of PMA2020.***

- We revised the result section to clarify.

***12- The heading of Table 02 should indicate that it contains bivariate analysis results.***

- Table 2 shows the results based on Analysis of Variances and Chi Square test between different round. We clarified this in the text and the explanation under the table.

***13- Pick between the adjectives, “highly” or “hugely” in Line 344.***

- We appreciate the reviewer picking this typo.

***14- The authors need to think a bit about what figures are reported versus those that are not for example. In Lines 357 and 358 they report the mean number of FP methods offered during each wave. They choose to report it as a range. However, given the mean methods available peaks in round 3 and then declines it may be better to just note which survey round had the highest mean of modern methods, as a precursor to a discussion.***

- We appreciate your recommendation. We revised the result section to follow your recommendation.

***15- Please carefully check the use of acronyms, as LAPM is sometimes written as LAMP (see Lines 360 to 362 as an example).***

- We appreciate you noticing this typo. We corrected the error across the text.

***16- In reporting figures, there is no consistency in the use of decimal places. For example, Table 03 uses one decimal place for all the figures. While Table 04 has some p-values reported with four decimal places. Do check with the journal, typically p-values less than 0.001 are simply reported as <0.001. Review the journal's guidelines and be consistent in the reporting of significant figures***

- We appreciate the reviewer for catching this problem. We fixed the inconsistencies in Table 3***.

  Discussion***

***17- The first few sentences in the paragraph beginning on Line 536 and going through Line 539 are confusing. Why not just state that enumeration areas do not equate to actual communities and that in reality, communities cross the demarcations produced in establishing EAs.***

- We followed the reviewer’s directions to clarify the mentioned limitation of this study.

***18- I think this section of the manuscript is where you bring up the shortcomings of DHS data which is what has typically been used for this type of analysis and how by using PMA2020 data you were able to avoid those shortcomings. As opposed to discussing those issues extensively within the introduction, as is currently the case.***

- We appreciate the reviewer’s recommendation. We agree on this point that the difference of DHS and PMA2020 methodology and instruments are important when we measure access using these resources. However, in the current study aimed to practically usePMA2020 results in the context of the DRC rather than discussing the survey. In addition, Choi et al. have published an article using DHS to measure access and have discussed the shortcomings of the instrument.

***Conclusion***

***19- Please review the paragraph beginning with Line 592. It is not entirely clear what is being proposed as a policy intervention to affect the supply chain of LAPMs.***

- We revised the conclusion to clarify.

***20- Additionally, the idea of FP supply is introduced rather late in the manuscript. Is this something the authors came across when researching the extant literature? Alternatively, did they find instances of other countries on the region/subcontinent struggling with supply as a policy issue? It may be something to bring up in the introduction then.***

-We revised the conclusion to clarify.
